# Analysis of Genetic Diversity and Phylogenetic Relationships of Wheat (*Triticum aestivum* L.) Genotypes Using Phenological, Molecular and DNA Barcoding Markers

**DOI:** 10.3390/genes14010034

**Published:** 2022-12-22

**Authors:** Mohamed A. El-Esawi, Mohamed M. A. Elashtokhy, Sahar A. M. Shamseldin, Enas M. El-Ballat, Ehab M. Zayed, Yasmin M. Heikal

**Affiliations:** 1 Botany Department, Faculty of Science, Tanta University, Tanta 31527, Egypt; 2 Genetics Department, Faculty of Agriculture, Zagazig University, Zagazig 44511, Egypt; 3 Botany Department, Women ’s College for Arts, Science and Education, Ain Shams University, Cairo 11566, Egypt; 4 Cell Study Research Department, Field Crops Research Institute Agricultural Research Center, Giza 12619, Egypt; 5 Botany Department, Faculty of Science, Mansoura University, Mansoura 35516, Egypt

**Keywords:** wheat, genetic variation, phenological traits, ISSR and SCoT markers, DNA barcodes

## Abstract

Wheat (*Triticum aestivum* L.) is a key food crop, accounting for approximately 765 million tons produced worldwide. The present study evaluated 16 wheat genotypes using 19 morphological and phenological traits, 16 molecular markers (Inter Simple Sequence Repeats and Start Codon Targeted; ISSR and SCoT) and *rbcL* and *matK* plastid gene barcoding. The 16 wheat genotypes showed significant genetic variation using the markers assayed. Cell plot of phenological parameters revealed significant differences among the 16-day-old seedlings of wheat genotypes at Z1.1 growth stage. Collectively, W2 genotype had the lowest shoot length (SL), length of first internodes (LFI) and leaf area (LA) values, while W8 genotype had the highest diameter of first internode (DFI) and LA values. Furthermore, W7 genotype had the maximum plant biomass (PB) and leaf width (LW) values. Geometric models grouped wheat kernels into “rounded” and “nearly elongated”. Estimates of heritability (H^2^) for these morphological characters ranged from 4.93 to 100%. The highest H^2^ values were recorded for root number (RN) (100%) followed by SL (88.72%), LFI (88.30%), LA (87.76%) and Feret diameter (86.68%), while the lowest H^2^ value was recorded for DFI (4.93%). Furthermore, highly significant genotypic and phenotypic correlations were also observed among those traits. Reproducible fingerprinting profiles and high levels of polymorphism (PPB%) of SCoT (95.46%) and ISSR (82.41%) were recorded, indicating that they are effective tools for detecting genetic variation levels among wheat genotypes. The informativeness of markers were measured through estimation of polymorphic information content (PIC), resolving power (RP) and marker index (MI). The RP and PPB% of SCoT were significantly higher compared to those of ISSR. Comparatively, the two molecular markers were effective for studying genetic diversity among wheat genotypes, but SCoT markers were more informative. Moreover, based on the two chloroplast DNA regions (*rbcL* and *matK*), *MatK* was found to be more reliable for differentiating among *T. aestivum* genotypes. Taken together, using all the studied attributes, a clear taxonomic relationship can be used to identify *T. aestivum* species and improve their pragmatic production and development.

## 1. Introduction

Bread wheat (*T. aestivum* L.) is among the most important food crops worldwide, used to make bread and straw for animal nutrition [1,2]. Varieties of *T. aestivum* ssp. *aestivum*, which are known for having naked kernels and producing the majority of commercial bread sold worldwide, are advantageous for milling in the bread and pasta industries in addition to the baking sector [3]. For fifty years, bread wheat was the most productive crop, and its production increased as a result of plant breeders’ efforts [4]. *T. aestivum* L. (2n = 6x = 42; genomes AABBDD) is a hexaploid species having A, B, and D ancestral genomes. The majority of bread wheat genes exist in the genome as triplicated homologous genes originated from the ancestor species. [5]. Despite having 21 pairs of chromosomes (3 homologous sets of 7 chromosomes in each of the A, B, and D sub-genomes), it genetically behaves as a diploid because homologous pairing is prevented by Ph genes [6]. It is also commonly referred to as “bread” or “soft” wheat and makes up approximately 95% of the wheat that is produced [5].

Wheat has a lengthy history of breeding that dates back centuries, as evidenced by the fact that it was discovered in the tombs of the pharaohs as well as in ancient civilizations and mentioned in the holy books [2,7]. Not just in Egypt but all throughout the world, wheat is the grain that is most crucial for ensuring food security. The largest wheat importer in the world, Egypt imports 12.5 million tons of wheat annually, which accounts for roughly one-fourth of all the food calories consumed there. Egypt’s wheat production in 2019–2020 was predicted to be 8.9 million tons, with 1.4 million hectares of wheat being planted there [8]. Egypt is expected to produce 23.8 million tons of cereal on average in 2021. The 2021 wheat production is anticipated to be 9 million tons, similar to last year and the five-year average [9]. 

A single genotype can be changed into one with desirable qualitative and quantitative traits using either a direct selection approach based on phenotypic values or target trait performance, or an indirect selection methodology, which can take several years. Indirect selection of target traits via linked molecular markers, which are unaffected by environmental variations and whose high frequency number and structural diversity allow for the sequencing, detection, and mapping of the targeted genes, is a recent development in biotechnology that aids in the solution [10]. As a consequence, this ground-breaking combination of modern molecular marker technology and conventional plant breeding procedures opens up new avenues for smart breeding [11]. 

Size, form, color, surface, growth habits, and other agronomic traits can all be visually distinguished using morphological markers. Morphological markers have been employed successfully in the breeding programs of many crops, including wheat, maize, rice, soybean, and tomato, for a very long time. They are still applicable for genetic and breeding applications. These markers’ primary disadvantages include their dominance in nature, sensitivity to plant development phases, pleiotropy, epistasis, and strong sensitivity to environmental changes [12]. Wheat’s morphological characteristics are readily measured with high accuracy, good quality, and comparatively high heritability for plant communities, which enhances screener effectiveness [13]. Prior work utilizing quantitative morphological variables to evaluate the morphological and phenological characteristics of wheat genotypes indicated significant morphological variability across the Triticum genotypes under study [14,15,16,17,18,19,20]. 

Geometric models for quantitative determination in seed images and shape description have been studied by scholars in recent years [3,21]. A seed cannot be cardioid, ovoid, and elliptical at the same time from a geometric perspective, and geometric shapes vary depending on the taxonomic group. In the instance of wheat, breeding that produces forms with higher yields may have an effect on the kernel shape. Furthermore, the quantification of seed shape could be an excellent element for validating the outcomes of automated inspection technologies as well as pinpointing the molecular causes of shape discrepancies [3]. 

Plant breeding initiatives must have genetic diversity in order to be effective and expand the gene pool. Heritability is occasionally used by breeding programmers to assess how successfully desired traits are handed down from parents to their offspring [22]. Estimation of heritability provides details on the level of genetic control over the expression of specific features and the accuracy of phenotypic predictions in determining breeding value [23]. The knowledge of correlations between important traits can make it easier to understand results and provide a foundation for the creation of breeding plans that are more effective. While genotypic correlation is the innate link between features, phenotypic correlation is the documented association between two values or traits. To produce results for range conditions, a plant breeder must understand the relationships between various yield ingredients [16]. 

Molecular markers enhance plant breeding, boost output, and accelerate time [24]. Effective DNA markers, such as SCoT and ISSR, are utilized to explore plant biodiversity [25]. Among the molecular markers, ISSRs were efficient for determining wheat germplasm intercorrelation [26,27]. Due to the great effectiveness of ISSR markers, just two primers were required to differentiate several of the wheat cultivars under study [28]. Additionally, SCoT polymorphisms are derived from the small, conserved area of plant genes that surrounds the ATG translation start codon [25]. The creation of core collections and the preservation of germplasm depend heavily on genetic markers. Utilizing molecular marker technologies to assess genetic variability, identify genotypes, and create DNA fingerprints, several researchers have identified the species of Triticum [18,19,29,30,31]. To make it easier to choose genotypes with higher diversity and improved performance, it is crucial to evaluate the germplasm’s genetic diversity.

The DNA barcode [32] is a genetic tract of short DNA sequences used in the identification of polymorphic plant species or as a genetic recognition approach that can be accurately identified by similar physical traits and chemical compositions [33]. Plastid sequences have traditionally been preferred to nuclear sequences in molecular plant taxonomy owing to their lower intra-molecular recombination [34]. Apart from traditional PCR-based markers, a short DNA sequence obtained from existing recognition sequences of the chloroplast genome can be utilized to classify plant genera and/or species in relation to orthologous databases [35]. *RbcL*, *matK*, *psbA-trnH*, and *ITS* barcodes are among the genes that have been studied for the identification and documentation of plant diversity. The chloroplast genome’s *rbcL* and *matK* gene loci have long been thought to be the standard for plant barcodes [32].

As a result, wheat breeders work hard to gain proper knowledge of the extent and genetic basis of variability throughout crucial characteristics in wheat genotypes. As a result, the current research aimed to assess the genetic variation in 16 Egyptian hexaploid wheat genotypes using morphological, phenological, molecular, and two gene barcoding attributes. This will also assist in the collection of data on the amount and nature of genetic variation that dominates yield expression, as well as the analysis of the related qualities in these wheat genotypes..

## 2. Materials and Methods

### 2.1. Plant Materials

The present research included 16 hexaploid wheat cultivars of *T. aestivum* L. The genotypes’ seeds were provided by Agricultural Research Center in Egypt, and their local names and pedigrees were reported in Table 1. The 16 cultivars were grown and the seedlings were collected for morphological analysis. Additionally, small young leaves were kept at −20 °C for molecular and barcoding analyses.

### 2.2. Experimental Design: Morphometric and Phenotypic Markers

The experiments were conducted in field condition following a randomized complete block design (RCBD) in three replications. The sowing was performed mechanically, using 15–20 grains per pot, depending on the genotype characteristics. A total of 45 16-day-old seedlings (three replications of each with 15 plants; 3 × 15) were taken at Z1.1 stage for each cultivar. According to Zadoks’ (Z) classifications of growth stages [36], there was one stage (one leaf (L1) on the main shoot and the second leaf (L2) appeared). Scan images from different genotypes were utilized to get quantitative measurements of the morphological traits. For shoot phenotypic traits: plant biomass (PB); shoot length (SL); length of first internodes (LFI); diameter of first internode (DFI) were measured. For leaf phenotypic traits: leaf area (LA) and leaf width (LW), and for root phenotypic traits: root number (RN), root maximum length (RL), root width (RW) and tip angle of root (TA) were calculated. For seed phenotypic traits: single seed weight (SW); seed area (SA); seed perimeter (SP); length of seed major axis (L); length of seed minor axis (W); aspect ratio (AR) (equals the ratio L/W); circulatory (circ.); roundness (round) and Feret diameter (Feret) were measured. Least Significant Difference (LSD) at 5% and analysis of variance were employed to compare the genotypes.

### 2.3. DNA-Based Molecular Genetic Diversity Analyses: SCoT and ISSR Markers

Young leaves of *T. aestivum* were utilized to extract DNA using the DNeasy plant mini kit. Later, until it was used for PCR amplification, the extracted DNA was stored at −20 °C. According to Collard and Mackill [25], 10 unique ISSR primers and 6 SCoT primers were utilized, as shown in Table 2. PCR amplifications were performed, according to Williams et al. [37], in a total volume of 30 µL, using 2 µL of each primer, 1 U of Taq polymerase, 2.5 mM of each deoxynucleotide, 25 mM MgCl_2_ and 25 ng of template DNA. The automated thermal cycle (Model Techno 512) used to perform the DNA amplifications consisted of 45 cycles, each lasting for 1 min at 94 °C, 1 min at 57 °C, and 2 min at 72 °C. After that, the reaction was kept at 72 °C for 10 min.

Utilizing a 1.5% agarose gel, ethidium bromide (5 g/mL), 1X TAE buffer, and 100 bp to 3 kb ladder markers, the amplified PCR products were separated electrophoretically. A tiny submarine gel unit (BioRad, Hercules, CA, USA) ran at 80 volts for approximately 30 min. The magnified bands were seen and captured in photos using a gel documentation technique under UV light (InGenius 3, Syngene, Frederick, MD, USA). Each amplification was repeated three times to ensure consistency of results. 

Only strong, clear, distinct and repeatable SCoT and ISSR amplified bands were recorded and converted to binary data. The percentage of polymorphism was calculated. Heterozygosity index (H) was used to evaluate the usefulness of ISSR and SCoT markers in identifying different *T. aestivum* genotypes. *H = 1 − Σ p_i_*^2^ [38] (*p*_i_ is the allele frequency for the *i*-th allele) and the polymorphic information content (*PIC* = 1 − *Σ p_i_*^2^ − *Σ Σ p_i_*^2^ *p_j_*^2^ [39], *p*_i_ and *p*_j_ are the population frequency of the *i*-th and *j*-th alleles) were calculated. Data analysis also included the calculation of the effective multiplex ratio, *E* = *n β* [40]; *β* = *n_p_/(n_p_* + *n_np_*), where np and p denote the nonpolymorphic and polymorphic fractions of the markers, so n_p_ and n_np_ denote their respective counting numbers. Average expected heterozygosity was calculated, *H.*av = Σ *H*_n_/*n*_p_ [40], where *H*_n_ is the heterozygosity of the polymorphic fraction of markers, and the summation is over all of the polymorphic loci *n*_p_. Marker index was also estimated, MI = E H.av [40]. Additionally, discriminating power was determined, D = 1-C [41], where the confusion probability is *C* = Σ *c*_i_ = Σ *p*_i_ Np_i_-1, where *C* is equal to the sum of all *c*_i_ for all of the patterns generated by the primer. Resolving power was estimated, Rp = Σ *I_b_* [42], where *I*_b_ or band informativeness is defined on a scale of 0–1 and is estimated as *I*_b_ = 1 − (2 × |0.5 − *p*|), where *p* is the portion of the observed band-containing samples.

### 2.4. DNA Barcoding Analysis: Plastid rbcL and matK Genes 

The reaction mixture for the PCR amplification of the two *rbcL* and *matK* genes contained 50 µL of ultra-pure water, 1 µL of Taq DNA polymerase, 0.2 mM dNTPs, 40 ng DNA, 15 mM MgCl_2_, 20 pcoml of each primer, and 20 pcoml of each primer. After a first denaturation cycle lasting 5 min at 94 °C, PCR amplification was conducted for 40 cycles. Each cycle consisted of a denaturation phase lasting for 30 s at 94 °C, an annealing step lasting for 30 s at 50 °C, and an elongation step lasting for 1 min at 72 °C. In the last cycle, the primer extension phase was prolonged to 7 min at 72 °C. Table 2 contained a list of the primers applied to the *matK* and *rbcL* genes for barcoding. The amplification products were electrophoretically separated in a 1.5% agarose gel comprising ethidium bromide (0.5 g/mL) in 1X TBE buffer at 95 volts. The molecular size reference used was a 100 bp DNA ladder. PCR products were seen and photographed.

All PCR products were purified using an EZ-10 spin column. Three volumes of binding buffer 1 were added into the PCR reaction mixture before the mixed solution was moved into an EZ-10 column and allowed to stand for 2 min at room temperature. After that, 750 L of wash solution were added into the column followed by centrifugation. After repeating the process to remove any remaining wash solution, the centrifuge was spun at 10,000 rpm for another minute. The column was put into a clean 1.5 mL microfuge tube, together with 50 L of elution buffer, and incubated there for 2 min before the pure DNA was kept at −20 °C.

Purified PCR products were sequenced using the Big Dye TM Terminator v3.1 Cycle Sequencing Kit in accordance with the manufacturer’s instructions on an automated sequencer, the ABI PRISM 3730XL Analyzer (Microgen Company, Moscow, Russia). DNA barcoding of sequences for the *rbcL* and *matK* genes was achieved using bioinformatical analysis. 

### 2.5. Statistical Analysis

For phenological and morphological markers, the mean and standard error of descriptive statistics were computed for phenological and morphological indicators of wheat genotypes. Before statistical analysis, the normality using Shapiro–Wilk at 0.05 level of all the phenotypic data was tested, and if necessary, the data were transformed. SPSS ver.22.0 was used to construct a one-way ANOVA. The JMP^®^ ver.16 cell plot was utilized for the graphical depiction (SAS Institute Inc., Cary, NC, USA, 2020–2021). Using the PLABSTAT program [43], the statistical analysis of phenotypic data was done in order to estimate variance and covariance, with genotypes being treated as fixed effects and the three replications being treated as random effects. By dividing genotypic variance (σ^2^G) by phenotypic variance (σ^2^p) and using the HERTI command of the PLABSAT software, heritability (H^2^) estimates were generated for each trait. The Genetic Coefficient of Variation (GCV) was also estimated. All tests had a 5% threshold of significance. Utilizing covariance analysis and the GENOT command of the PLABSTAT software, the phenotypic and genotypic correlation coefficient for each trait was calculated. JMP^®^ ver.16 was used to create a constellation plot of hierarchical clustering using Ward’s approach to distribute 16 Egyptian wheat cultivars based on phenological and morphological characteristics. 

For molecular marker analysis, the online marker efficiency calculator, or iMEC (https://irscope.shinyapps.io/iMEC/, accessed on 15 August 2022) was used to determine the ISSR and SCoT polymorphism indices [44]. The genetic diversity and similarity matrix of 16 Egyptian wheat genotypes was expressed by hierarchical co-clustering using Ward’s approach and heatmap based on combined marker analysis (ISSR and SCoT) polymorphism using JMP^®^ ver.16. 

In order to summarize the data and better understand the findings, Principal Component Analysis (PCA) was performed to investigate the contribution of the combination of molecular markers (ISSR and SCoT) as well as individual morphological and molecular features to overall variability. The Guttman–Keiser criterion was used to decide how many components were taken for additional examination. Components with an Eigen value greater than 1 were kept for further examination. Genotypes were grouped using a clustergram based on how closely they shared the features that were put to the test, as well as how closely these traits correlated. By using the Ward method of clustering, the similarity matrix was created. JMP^®^ ver.16 was used to conduct the two analyses.

The sequences generated by each *rbcL* and *matK* marker were aligned in DNA barcoding analysis using multiple sequence alignment (multalin) http://multalin.toulouse.inra.fr/multalin/, accessed on 15 August 2022). A homology search of *rbcL* and *matK* sequences was analyzed using the BLAST program (http://www.ncbi.nlm.nih.gov/BLAST, accessed on 15 August 2022). The resolution of each locus was determined by the Maximum Likelihood (ML) and the Neighbor Joining (NJ) trees built using the UPGMA algorithm by MEGAX software. The bootstrap methods with 1000 replications were used to test the reliability branching in the ML and NJ analyses. Tamura-3 parameter was calculated for loci [45]. Using DnaSP 6.10.01, the total alignment length (bp), the total number of matrix cells, the percentage of missing data, the number of parsimony informative sites (PIC), the number of variable sites, and the average G+C contents in each region were used to describe the genetic variability of each marker.

## 3. Results and Discussion

### 3.1. Phenological Markers

#### 3.1.1. Phenological, Morphological and Geometric Traits Characterization

The interplay of phenotyping and genotyping, as well as their interactions with each other and the environment, affect wheat (*T. aestivum* L.) crop performance. For example, in order to achieve maximal seed count and size (potential yield), wheat must maintain and improve biomass and flowering during ideal seasonal conditions [46]. Quantitative traits are complicated because of the involvement of multiple genes and their interrelations with one another and the environment. Plant architecture and phenology are two quantitative characteristics that contribute significantly to wheat adaptation [47]. 

**Figure 1:**
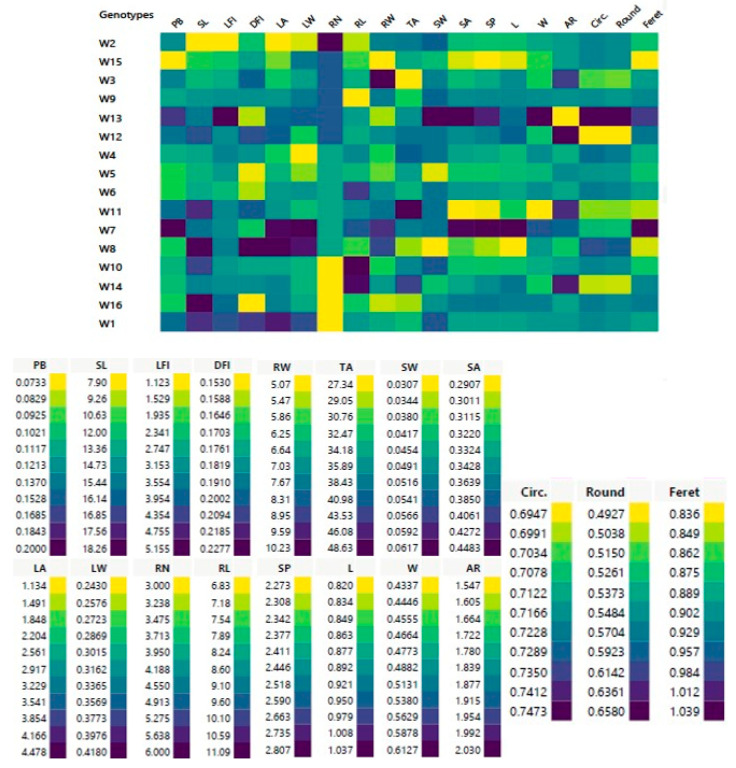
Cell plot of 19 morphometric traits of shoot, leaf, root and seed of 16 studied Egyptian *T. aestivum* genotypes. Data represents means of three replicates; PB = plant biomass (g); SL = shoot length (cm); LFI = length of first internodes (cm); DFI = diameter of first internode (cm); LA = leaf area (cm^2^); LW = leaf width (cm) RN = root number; RL = root maximum length (cm); RW = root width (cm); TA = tip angle of root (°); SW = single seed weight; SA = seed area; SP = seed perimeter; L = length of seed major axis; W = length of seed minor axis; AR = aspect ratio; Circ. = circulatory; Round = roundness; Feret = Feret diameter.

The results of morphological traits showed tremendous variations among Z1.1 stage of 16-day-old wheat genotypes (Figure 1 and Table 3). These variations are essential for developing new cultivars with distinct morphological and phenological traits. This study tested 19 quantitative wheat traits; the plant biomass (PB) of all genotypes was between 0.07 and 0.20 g with a mean value of 0.12 g. For shoot phenotypic parameters, the shoot length (SL) of the W16 genotype was the longest (18.26 cm) while the shortest (7.90 cm) was noted in the W2 genotype with an average value of 14.73 cm. Length of first internodes (LFI) values were between 1.12 and 5.16 cm with a mean value of 3.15 cm. Diameter of first internode (DFI) values varied from 0.15 to 0.23 cm with an average value of 0.18 cm as recorded (Table 3). In accordance with Lukovi et al. [17], the KG-1/6 genotype (78.5 cm) had the lowest average stem height of 14 winter wheat (*T. aestivum* ssp. *vulgare* L.) genotypes at three locations across Serbia at the yield stage, while the KG-162/7 genotype had the highest (102.3 cm). Pobeda genotype had the largest internode diameter (4.0 mm), while (KG-191/5-13) genotype had the smallest diameter (3.5 mm). The average length of the first internode was 3.5 cm (KG-1/6) to 5.1 cm (KG-191/5-13).

In this investigation, the leaf area (LA) and width (LW) were essential traits and these traits were in the ranges of 1.13–4.48 cm^2^ and 0.24–0.42 cm, respectively. The widest leaf area was from the W8 genotype and the smallest was from the W2 genotype, respectively (Table 3). Collectively, the W2 genotype had the lowest SL, LFI and LA values, while the W8 genotype had the highest DFI and LA values, and W7 genotype had the maximum PB and LW values.

Wheat selection and breeding programs have largely ignored the impact of root traits on aboveground traits and yield [48]. Recognizing the morphology of the root system in bread wheat is essential for identifying root traits in order to produce genotypes with enhanced resource uptake and tolerance to harsh conditions [49]. Some studies have examined the phenology and morphology of large collections of wheat germplasm [50]. Root phenes are root system morphological traits that comprise root numbers, length, angles, and different root diameter categories [51]. 

In the current study, root maximum length (RL), root width (RW), and tip angle of root (TA) indicated significant variations across all wheat genotypes, as shown (Figure 1 and Table 3). W2 genotype had the highest RN (6.00), while W1, W10, W14, and W16 genotypes were recorded as (3.00). The average RL of all genotypes was 8.60 cm, whereas the highest was in W10 (11.09 cm) and the lowest was found in W9 (6.83 cm); similar to Chen et al. [49], who discovered that the total RL at the Z2.1 stage, 35 days after wheat plant transplanting varied from 670 to 3538 cm, with an average of 1937 cm. Among the 184 genotypes, 79 genotypes had RL values between 1000–2000 cm per plant, 6 genotypes had RL values less than 1000 cm per plant, and 5 genotypes had RL greater than 3000 cm. Other studies of Waines and Ehdaie [52] and Fang et al. [53] have confirmed modern wheat varieties with smaller root systems than older ones as an unintended result of breeding for improved grain yield.

Root length and number, emergence and tip angles, rooting depth and width, convex hull area, and root mass center are all architectural characteristics [54]. The architectural characteristics of seedling roots also significantly affect the phasic development. Remarkably, a larger root system is linked with delayed maturation and, consequently, extended grain filling, most likely due to improved nutrient and water uptake for photosynthesis [55]. In this investigation, the average RW of all genotypes was 7.03 cm, whereas the highest was in W3 genotype (10.23 cm) and the lowest was found in W15 genotype (5.07 cm). TA average of all genotypes was 35.89°, the largest angle was in W11 genotype (48.63°), and the smallest acute angle (27.34°) was recorded in W3 genotype (Figure 1 and Table 3). Furthermore, in the W3 genotype, a smaller tip angle of seminal roots was associated with a wider root system (RW). Our findings were consistent with those of Xie et al. [56], who studied 226 recombinant inbred lines (RIL) of 13-day-old seedlings obtained from a cross between *T. aestivum* ‘Forno’ (wide and small root system) and *Triticum spelta* ‘Oberkulmer’ (narrow and large root system).

Tetra-and hexaploid species have larger grains. Morphological variations of wheat kernels from various genotypes were recorded [57]. In this study, modern shape analysis methods depart from the coordinate of the points in the outlines of a figure based on descriptive parameters for seed phenotypic and geometric traits (i.e., single seed weight (SW); seed area (SA); seed perimeter (SP); length of seed major axis (L); aspect ratio (AR); length of seed minor axis (W); circulatory (Circ.); roundness (Round); Feret diameter (Feret). On average, SW of all genotypes was 0.05g and W2, W3, W4, W6, W7, W9, W10, W11, W12 and W16 genotypes shared the same seed weight (0.05 g). The average of SA and SP values were 0.34 cm^2^ and 2.45, respectively (Table 3). The genotypes W7 and W13 had maximum SA and SP values whereas the minimum values were obtained in W11 and W15 genotypes, as represented (Figure 1 and Table 3). In addition, the average Feret diameter of all wheat genotypes was 0.90 cm, whereas the highest was in W7 genotype (1.04 cm) and the lowest was found in W8 and W15 genotypes (0.84 cm) as scored (Table 3).

**Figure 2:**
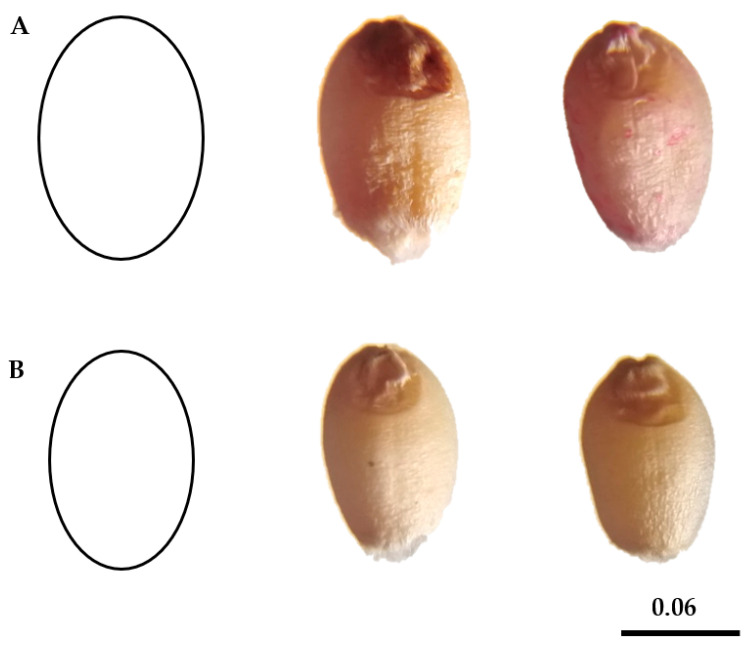
Two geometric models of wheat kernels are (**A**): an ellipse of aspect ratio = 1.8 (more rounded) for W2 and W16 genotypes (from left to right) and (**B**) an ellipse of aspect ratio = 2.03 (more nearly elongated) for W12 and W14 genotypes (from left to right). Bar corresponds to 0.06 cm.

Recent developments of comparing the shapes of two-dimensional figures rely on artificial visions and incorporate the point coordinate in a figure’s profile. Using these virtues, the application of algorithms series allows for the calculation of several traits for each figure, including: its shorter and longer diameters, centroid, perimeter, area and circularity index [3,58,59]. Kernels of W13 genotype had a lower AR (1.55) than the other genotypes, while kernels of W12 genotype had a higher AR (2.03). However, the kernel of W13 genotype had higher Circ. and Round values and in the case of W12 genotype had lower Circ. and Round values (kernels are more elongated). Different models for quantifying seed shape were proposed based on variations in roundness and circularity, as well as a visual examination of kernel shape. According to the outcomes of the two models, wheat kernels are divided into two groups based on their shape: “rounded” kernels and “nearly elongated” kernels, as shown (Figure 2 and Table 3).

In the present study, an AR = 1.8 ellipse was designed to fit the shape of the W2, W10, W15, and W16 genotypes (more rounded), and an AR = 2.03 ellipse was designed to fit the shape of the W12 and W14 genotypes (more near elongated). Our findings agreed with those of Martín-Gómez et al. [3], who concluded that kernels of modern varieties (hexaploid common wheat) with an ellipse of AR = 1.8 were made to integrate the shape of *T. aestivum* ssp. *aestivum* Zebra (TaaZ).

#### 3.1.2. Availability and Hereditability of the Wheat Genotypes 

Heritability, a measure of phenotypic variation, plays a key role in crop breeding [60]. A crop population’s phenotypic and genotypic variances (PCV and GCV) are critical for successful plant breeding. They are critical parameters for determining the impact of the environment on the genotype’s performance [61]. The examination of mean sum of squares values revealed that genotype variance was significant for all 19 morphometric and phenological traits, indicating that genetic variation existed among the 16 *T. aestivum* genotypes, as shown in Table 4. Mean squares of TA along with other determinants were the highest among different genotypes. The character (TA) had the highest genotypic and replication coefficient of variation (GCV and RCV) (20.017 and 8.2029%), respectively. Our findings were in agreement with that of Lukina et al. [62]. Furthermore, Hossain et al. [63] discovered that the PCV of all traits was greater than the GCV and that their values were closer to each other among 25 genotypes of spring wheat cultivar. Analogous findings were recorded by Varsha et al. It was found by [16] that the biological yield per plot had the highest GCV value (18.09%), while the GCV and PCV showed the desired values for the remaining traits, such as the plant height of 98 wheat genotypes, which were comparatively lower than (10).

High heritability demonstrated that classification for these characters would be efficient, as it would be less influenced by environmental effects [64]. The simpler are the selection procedures, the higher are the heritability estimates [65]. In this investigation, heritability (H^2^) ranged from 4.93% to 100%. The highest H^2^ values were recorded for RN (100%), then came SL (88.72%), LFI (88.30%), LA (87.76%), and Feret (86.68%), while the lowest was recorded in DFI (4.93%) (Table 4). The traits TA, SL, RN, and LA showed high GCV and heritability. Sharma and Garg [66] reported similar results in wheat (*T. aestivum* L.) crosses grown in variable natural and salinity conditions. Hossain et al. [63] also discovered that the estimated heritability for various physiological and yield contributing traits of 25 spring wheat cultivar genotypes varied from 29.41% to 97.44%.

#### 3.1.3. Phenotypic and Genotypic Correlation Coefficients 

Genotypic correlation values are used for further analysis because correlations must be made in light of their genetic behavior. Trait genetic relationships can result from the pleotropic effects of a gene, chromogema, the linkage of two genes and regimental affiliation, or environmental influences [67]. In this investigation, the genotypic (r_g_) and phenotypic (r_p_) correlations were estimated to determine the relationships between various quantitative characters at the phenotypic level (Table 5). The results showed that the (r_p_) was smaller than (r_g_) correlation among all phenotypic traits. These correlation results confirmed the cell plot phenological data of different studied genotypes. Among the shoot phenotypic scores, LFI had the maximum correlation with SL, LA, LW, RN, SA, SP, L, W, Circ., Round and Feret, in which LFI was significantly positive correlated with SL (phenotypic correlation (r_p_) = 0.73 **, *p* < 0.01, (genotypic correlation (r_g_) = 0.78+) as shown in W2 genotype. Among the leaf phenotypic scores, LA was significantly positive correlated with SL and LFI (r_p_) = 0.80 ** and 0.66 **, *p* < 0.01 and (r_g_) = 0.84+ and 0.75++, respectively, as shown in W2 genotype. In addition, LA was significantly positive correlated with LW (r_p_) = 0.75 **, *p* < 0.01 and (r_g_) = 1.51+. Among root phenotypic scores, RN was significantly negative correlated with SL and LA (r_p_) = −0.67 **, *p* < 0.01 and −0.45 and (r_g_) = −0.72++ and −0.48 ++, respectively. Along with, RL was negatively correlated with RN (r_p_) = −0.61 *, *p* < 0.05 and (r_g_) = −0.74+ as recorded in W10 genotype (Table 5). 

All the seed geometric scores (SW, SA, SP, L, W and Feret) were highly significant positive correlated with PB, SL, LFI and LA, while they were significantly negative correlated with AR. In addition, AR was significantly negatively correlated with W, Circ. and Round (r_p_) = −0.69 **, −1.00 ** and −0.99 **, *p* < 0.01 and (r_g_) = −0.64++, −1.00++ and −1.00++, respectively, as recorded in W13 genotype. In contrast, W was significantly positively correlated with Circ., Round and Feret (r_p_) = 0.67 **, 0.73 ** and 0.76 **, *p* < 0.01 and (r_g_) = 0.62++, 0.69++ and 0.81++, respectively, as recorded in W13 genotype. In addition, Feret was significantly positively correlated with PB, SA, SP, L and W(r_p_) = 0.76 **, 0.95 **, 0.99 **, 0.95 ** and 0.76 **, *p* < 0.01 and (r_g_) = 1.07++, 0.97++, 0.99++, 0.97++ and 0.81++, respectively, as recorded in W7 genotype. Circ. had the highest positively correlation with Round, while both had a negative correlation with AR as shown in the W12 genotype as scored in (Table 5).

The co-efficient of variation (CV%) was low for all parameters, indicating that the difference between each individual and another was minimal, indicating that the results for these traits were more acceptable [68]. In this investigation, the r_p_ was generally lower than the r_g_ for all 19 phenotypic traits of 16 wheat genotypes, and PB was positively correlated with all traits except DFI and AR. According to Ahmed et al. [69], seedling length (SL) had a negative correlation with DTW and highly significant and positive correlations with the five leaf wilting scores. SL can be used as a predictor of drought tolerance.

Mukherjee et al. [70] investigated genotypic and phenotypic coefficients of variation in bread wheat and revealed high coefficients of variations of spikes per plant. Likewise, Sarkar et al. [71] concluded that spikelets per spike, plant height, and yield per plant of 37 wheat lines had higher heritability, genetic advance, and phenotypic coefficient of variation.

#### 3.1.4. Hierarchical Co-Clustering Analysis Based on Morphological and Phenological Markers

**Figure 3:**
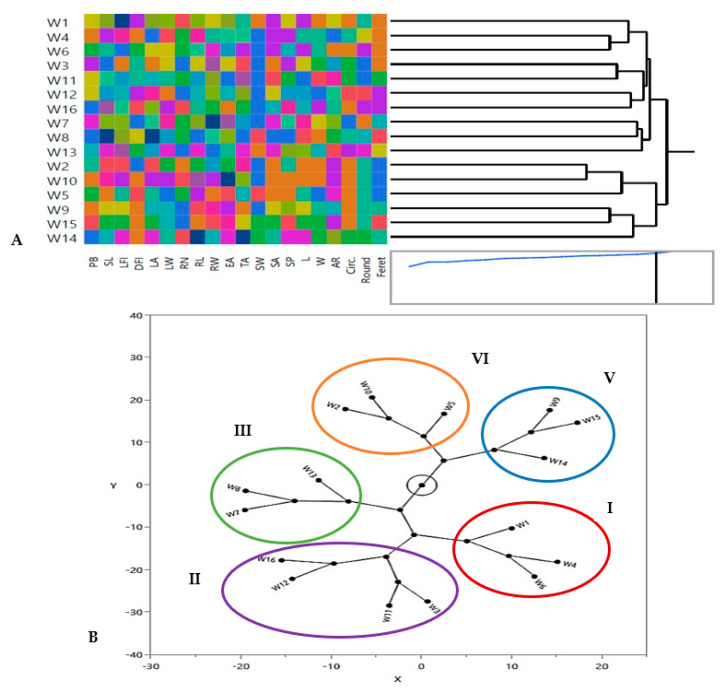
Clustering multivariate analysis using Ward’s method for distribution of 16 Egyptian *T. aestivum* genotypes based on the phenological and morphological markers by using (**A**) hierarchical co-clustering dendrogram and heatmap; row clusters were obtained at genotype level, whereas the column cluster were recorded at trait or marker level; (**B**) Constellation plot from hierarchical cluster analysis showing five clusters (Clusters I, II, III, IV and V represent clusters as red, violet, green, orange and blue oval shapes).

Selection of genotype groups is primarily influenced by the breeding program’s objectives [72]. Multivariate analysis based on hierarchical co-cluster analysis and constellation plot using Ward’s method was performed to identify the phenotypic diversity of the 16 wheat genotypes due to differences in morphological and phenological traits and grouped the genotypes into 5 distinct clusters as represented in Figure 3A,B. A co-cluster matrix consisted of 5 rows (genotypes) and 4 column clusters (traits). Genotypes of cluster I were W1, W4, and W6 which had similar seed geometric traits. Additionally, cluster II had the largest number of genotypes (W3, W11, W12 and W16), which had similar RL and RW values. Cluster III consisted of W7, W8 and W13 genotypes, which had similar leaf traits (LA and LW), while genotypes of cluster IV were W2, W10 and W5, which had similar TA of root architecture and cluster V had W9, W15 and W14 genotypes which shared the same seed geometric traits (Figure 3A,B). In the same trend, Mohi-Ud-Din et al. [72] concluded that, using Ward’s method, the genotypes of different hierarchical row clusters exhibited variable alterations in the effectiveness of analyzed wheat seedling characteristics. 

### 3.2. Molecular Genetic Diversity Analyses: SCoT and ISSR Markers 

Molecular characterization is now a valuable tool for assessing variation in plant genetic resources. Polymorphism is also considered a useful selection tool in monitoring alien genome introgression in wheat breeding programs. In the present study, two marker systems (ISSR and SCoT) were used to investigate the genetic variability of wheat genotypes. The polymorphism level and the discriminating capacity of these markers are summarized in Table 6. 

#### 3.2.1. ISSR Markers

Using 10 ISSR primers, out of 79 scorable amplified bands (TAB), 64 were PB (polymorphic) and 15 were MB (monomorphic) with an average frequency of bands (MBF) 0.49. Their molecular size ranged from 84 to 4039 bp. The percentage of polymorphism (PPB) ranged from 57% (ISSR-841) to 100% (ISSR-857, ISSR-814 and ISSR-840) with an average of 82.41%. Out of 56 UB (unique bands), the maximum bands (9) were recorded in ISSR-857 primer, which achieved the highest %PPB as 100% (Table 6). The %PPB value based on ISSR markers was variable in previous reports. El-Aref et al. [73] found that the polymorphism percentage has an average of 64.95% among wheat varieties. In addition, a high P% was reported in wheat genotypes using ISSR markers [74,75]. 

Polymorphism information content (PIC) plays a key role in genetic variation analysis [76,77]. Furthermore, several parameters were calculated in this study to categorize the informativeness of the primers under consideration as PIC, heterozygosity index (H), average expected heterozygosity (H. av), marker index (MI), discriminating power (D), effective multiplex ratio (E) and resolving power (Rp). In this investigation, the patterns of ISSR-807, ISSR-825 and ISSR-827 primers recorded the highest values of 0.50 and 0.37 for H and PIC, respectively, whereas ISSR-826 primer noted the lowest values for H (0.35) and PIC (0.29). According to Botstein et al. [39] and Ramadugu et al. [78], markers with a PIC value of 0.25 to 0.50 provide valuable information for genetic variation studies. In the present study, the mean PIC value for ISSR markers was 0.35, with a range of 0.29 to 0.37. As a result, all of the primers tested in this study were found to be effective.

The PIC and Rp prepared a degree of marker system capacity, assisting in the study of primer efficiency in the evaluation of genetic diversity [79]. In this investigation, E values ranged from 0.8 in ISSR-814 primer to 7.7 in ISSR-826 primer. Additionally, the patterns of ISSR-807, ISSR-810, ISSR-835, ISSR-841, ISSR-825, ISSR-826 and ISSR-827 primers revealed the highest values (0.02) of MI. In addition, D values ranged from 0.40 in ISSR-826 primer to 0.93 in ISSR-840 primer, whereas Rp values ranged between 1.2 in ISSR-814 primer and 4.9 in ISSR-857 primer (Table 6). 

#### 3.2.2. SCoT Markers

Using 6 SCoT primers, however, revealed 64 amplified fragments with molecular sizes ranging from 177 to 3658 bp and an average of 10.70 bands for each SCoT primer, with 61 fragments being polymorphic with 54 UB and the remaining 3 fragments being monomorphic (Table 6). The %PPB ranged from 82% (SCoT 2) to 100% (SCoT 1, SCoT 4, SCoT 5, and SCoT 6) with a mean of 95.46%. Furthermore, the %PPB of the studied SCoT marker in this study was greater than that of Khodaee et al. [80], who discovered that the SCoT primers produced 162 amplified fragments. The majority of them (90.74%) were polymorphic among Iranian *Aegilops triuncialis* accessions. Similarly, Shaban et al. [31] confirmed that the primers SCoT3, SCoT5, SCoT10, and SCoT12 revealed 100% polymorphism and identified a significant degree of variation among wheat germplasm.

In this investigation, as for the frequency of the bands, MBF was scored as 0.49. SCoT 1, SCoT 4 and SCoT 6 primers recorded the lowest values of H (0.40) and PIC (0.30), while primers SCoT 2, SCoT 3 and SCoT 5 showed the highest values of H (0.50) and PIC (0.40), respectively. Our results in accordance with Nosair [30] found that the %PPB revealed by the different primers varied from 100% for SCoT (7, 14, 24, 28, 35 & 46) primer to 90% for SCoT11 primer. PIC values ranged from 0.40 (SCoT 24) to 0.62 (SCoT 35), with an average value of 0.52 per primer. In this study, SCoT 1 primer had the lowest E value (2.6), while SCoT 2 primer had the highest E and MI values (6.3 and 0.02). In contrast, SCoT 2 primer had the lowest D value (0.7), whereas the highest D value (0.9) was recorded in SCoT 1, SCoT 3, SCoT 4 and SCoT 6 primers. In addition, Rp values ranged from 3.3 to 7.1 in SCoT 1 and SCoT 5 primers, respectively, as recorded in (Table 6). In the same trend, Shaban et al. [31] reported that the ISSR and SCoT markers have PIC values of 0.61 and 0.62, respectively. SCoT markers had the highest levels of Rp, MI, and polymorphism percentage.

#### 3.2.3. ISSR and SCoT Analysis

ISSR and SCoT markers have been shown to be useful in genetic variation studies due to their excellent reproducibility and strength for polymorphism detection [26,81]. In this study, a total of 143 amplified bands or fragments (125 polymorphic and 18 monomorphic) resulted from combined ISSR and SCoT primers of wheat genotypes with an average of 9.53 bands per primer. Table 6 shows that the %PPB was as high (88.94%), with 15 unique polymorphic bands with an average of approximately 1 for each primer, 110 non-unique polymorphic bands (NUB) with an average of 7.33, and 18 monomorphic bands (MB) with an average of 1.2. The efficiency of both ISSR and SCoT markers is estimated in this study using parameters, such as PIC, MI, and Rp. In fact, SCoT markers were more effective than ISSR markers at detecting polymorphisms, detecting 95.46% versus 82.41% polymorphisms. The average values of H, PIC, E, MI, D, and RP of ISSR primers were 0.45, 0.35, 4.04, 0.01, 0.74, and 2.92, respectively, while the average values of H, PIC, E, MI, D, and RP of SCoT primers were 0.40, 0.30, 3.9, 0.01, 0.9, and 5.4, respectively. According to our findings, the mean values of %PPB and Rp for SCoT primers were higher than those for ISSR primers, demonstrating the great potential of SCoT markers in assessing genetic diversity and determining relationships among wheat genotypes. These findings support the findings of Nosair [30] and Shaban et al. [31], who concluded that SCoT markers were more informative in studying genetic diversity among wheat cultivars. According to Khodaee et al. [80], the most relevant indices for measuring marker efficiency were Rp and MI, though the ISSR had a higher resolution than the SCoT markers among Iranian *A. triuncialis* accessions, contradicting our findings.

The present study showed that the ISSR-857 and SCoT 4 primers were the most informative and had the greatest potential of the primers tested. ISSR primers resulted in 56 amplified UB, while SCoT primers resulted in 54 amplified UB. Interestingly, the ISSR-857 primer revealed the most UB (9 bands), while the SCoT 4 primer revealed the least UB (11 bands). The HB-10 and SCoT 1 had the highest values for all parameters studied. Gowayed and Abd El-Moneim [27] found that the HB-10 primer revealed the highest value of P% of 96% and the SCoT 1 primer revealed the highest value of P% of 92.3% of some Egyptian wheat genotypes. 

The similarity coefficient among the studied wheat genotypes using combined data of ISSR and SCoT was recorded in (Table 7). The pairwise comparisons between the tested genotypes were utilized to estimate the genetic similarity matrix coefficients. The highest similarity index (0.89) was found between W10 (GEMMIZA 9) and W12 (GEMMIZA 11) genotypes, while, W4 (SHANDWEEL 1) and W6 (SAKHA 95) had the lowest similarity index (0.54), reflecting a wider genetic diversity between them. In addition, W3 (SIDS 14), W8 (GIZA 168), W9 (GEMMIZA 7) and W10 (GEMMIZA 9) genotypes had the highest similarity value with W1 (SIDS 1) genotype. 

#### 3.2.4. Multivariate Analysis Based on ISSR and SCoT Combined Data: Hierarchical Co-Clustering Analysis 

**Figure 4:**
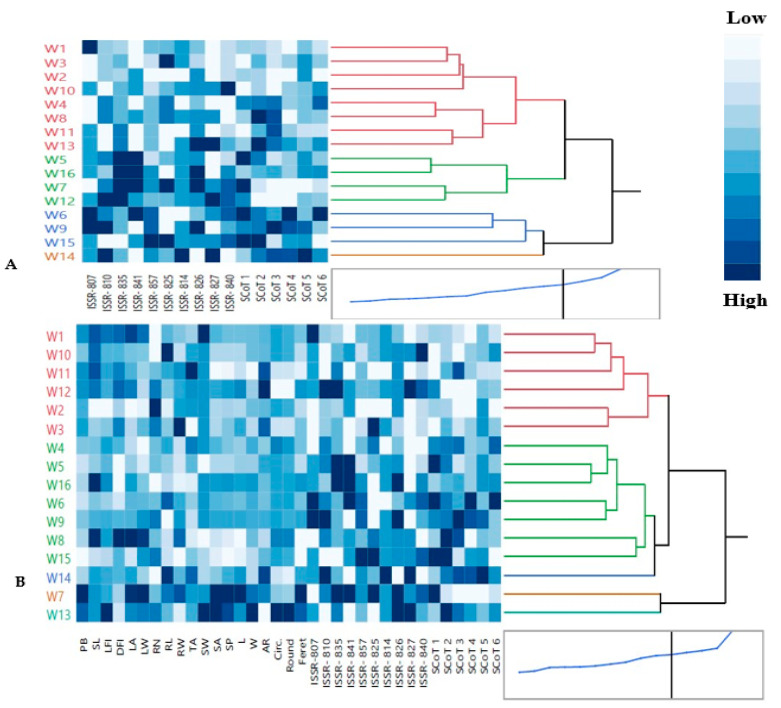
Hierarchical co-clustering dendrogram and heatmap using Ward’s method illustrating the distribution of 16 Egyptian *T. aestivum* cultivars based on: (**A**) the polymorphism values of combined molecular markers (10 ISSR and 6 SCoT); (**B**) 19 morphological and 16 molecular markers. Correlation levels are colored dark blue for high intensities and white for low intensities. Row clusters were obtained at genotype level, whereas the column cluster were recorded at trait or marker level.

Based on a hierarchical co-clustering dendrogram and heatmap pattern derived from a combined analysis of SCoT and ISSR data (Figure 4A), the 16 wheat genotypes were classified into 4 main clusters. A co-cluster matrix was consisted of 4 row (genotype) and 2 column clusters (traits or markers). The first cluster I was the largest group included 8 genotypes: W1 (SIDS 1), W3 (SIDS 14), W2 (SIDS 12), W10 (GEMMIZA 9) in a sub-cluster, which had low intensities and weak correlation to each other through SCoT primers and moderate correlation through ISSR primers and also, W4 (SHANDWEEL 1), W8 (GIZA 168), W11 (GEMMIZA 10) and W13 (GEMMIZA 12) genotypes in another sub-cluster, which positively correlated through SCoT2 and SCoT3. Cluster II contained, W5 (SAKHA 94), W16 (MISR 3), W7 (GIZA 171) and W12 (GEMMIZA 11), which had high intensities (dark blue) and a highly positive correlation through ISSR-810, ISSR-835, ISSR-841, ISSR-826 and SCoT 1. Additionally, genotypes of cluster III were W6 (SAKHA 95), W9 (GEMMIZA 7), W15 (MISR 2) which had high and moderate positive correlation through ISSR-807, ISSR-810, ISSR-857, ISSR-825, ISSR-827 and all SCoT primers. Cluster IV had only W14 (MISR 1) genotype. The dendrogram derived from SCoT revealed the best clustering pattern in this study. Our findings were in concordance with those of Etminan et al. [29]. These studies indicate that these molecular markers are crucial criteria for evaluating wheat genotypes genetic diversity. Similarly, Carvalho et al. [74] used ISSR markers to analyze 48 wheat cultivars and discovered that most cultivars from the same botanical variety were grouped together. El-Aref et al. [73] discovered that the ISSR markers were successful in distinguishing wheat varieties based on their ploidy level and location, with tetraploid varieties and hexaploid varieties grouped together in one group.

### 3.3. Relationships among Morphological and Genetic Attributes

#### 3.3.1. Hierarchical Co-Clustering Analysis 

The dendrogram reflected the differences in genetic diversity within the 19 phenological and molecular markers (ISSR and SCoT) levels and discriminated among the wheat genotypes as represented in Figure 4B. The 16 wheat genotypes were divided into 4 main clusters. A co-cluster matrix consisted of four row (genotype) and six column clusters (traits or markers). Cluster I included six genotypes: W1 (SIDS 1), W10 (GEMMIZA 9), W11 (GEMMIZA 10), W12 (GEMMIZA 11), in a sub-cluster 1 and also, W2 (SIDS 12) and W3 (SIDS 14) genotypes in another sub-cluster 2. Furthermore, Cluster II had W4 (SHANDWEEL 1), W5 (SAKHA 94), W16 (MISR 3), W6 (SAKHA 95), W9 (GEMMIZA 7), W8 (GIZA 168), W15 (MISR 2) genotypes. Cluster III had W14 (MISR 1) genotype. Additionally, genotypes of cluster IV were W7 (GIZA 171) and W13 (GEMMIZA 12). Multivariate compound similarity analysis is commonly used to show additional data about plant breed genetic variance, which is detailed in heatmaps [82]. Correlation analysis assists in determining impactful traits for indirect selection of superior genotypes. Concerning the heatmap correlation, cluster I genotypes had a strong correlation between leaf (LA and LW) and root parameters (RN, Rl and RW). Moreover, there were moderate correlations of seed parameters (TA, SW, SA, SP, L, W, AR, Circ., Round and Feret) among genotypes of cluster II especially in sub-cluster 1, while the strongest correlations were found between cluster IV genotypes (W7 and W13). 

#### 3.3.2. Principal Component Analysis (PCA)

The principal component analysis (PCA) technique was used to analyze object diversity in terms of quality traits and group them according to the similarity hierarchy [83]. As a result, correlation and principal component analyses assist breeders in genetically improving traits with low heritability, such as yield, particularly in early generations, through indirect selection for traits effective on this [84].

**Figure 5:**
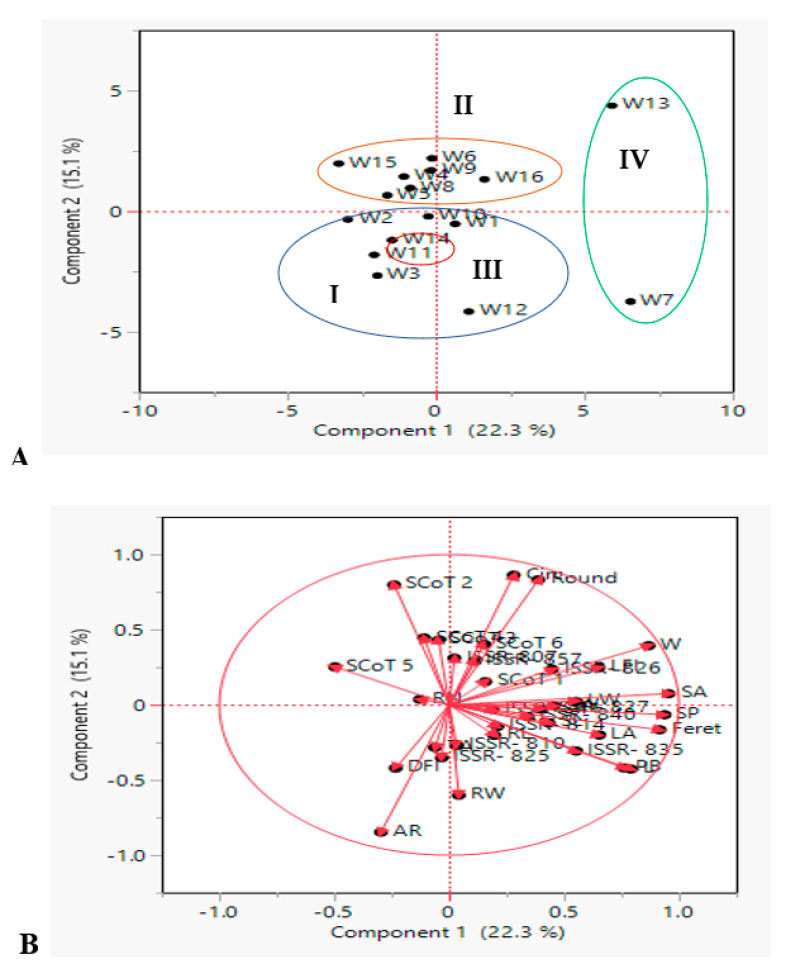
Principal component analysis (PCA) illustrating the genetic diversity expressed by the grouping of 16 Egyptian *T. aestivum* cultivars based on the analysis of 19 morphometric traits, 10 ISSR and 6 SCoT primers polymorphism (**A**) scatter plot analysis showing four clusters (Cluster I, II, III and IV represent as blue, orange, red and green oval shapes, respectively); (**B**) the interrelationship among all measured morphological and molecular marker parameters. The dots are genotypes and the vectors (red arrows) are parameters.

PCA was also performed to support the results of the hierarchical clustering analysis (Figure 5A), which was based on their genomic constitution and 19 phenological traits. PCA was used to analyze the average data. PCA reflects the significance of the largest contributor to total variation along each differentiation axis. PCA revealed similar groups, correlating the results of neighbor-joining clustering. In a PCA scatterplot, variation in the 16 wheat genotypes described by the first 2 PCs per cluster, ranged from 15.1 to22.3% of the total variation as shown in Figure 5A. For these dendrogram co-clusters, PCA produced mostly congruent results. The studied genotypes were also grouped into four clusters: cluster I (blue group) included six genotypes, cluster II (orange group) had 7 genotypes, cluster III (red group) had one only genotype which were distinct and well supported and cluster IV (green group) had two genotypes. Furthermore, objects with similar ordinates were more similar than those with dissimilar ordinates.

In the system of two first components, length of vector and cosine of angle were used for discrimination of wheat genotypes were shown in Figure 5B. Circ. and round are the longest vectors, and the small angle between these vectors proves a significant strong positive correlation between these traits. On the other side, circ. and round traits had a strong positive correlation with each other and a negative correlation with AR. Moreover, vectors for PB and some seed geometric parameters (W, SA, SP, Feret, L) showed the strong discrimination power of these parameters and might be important for the evaluation of wheat genotypes. PB created 90 degrees with AR, the same pattern was shown also between W and AR in which they were not likely to be correlated. These results confirmed the cell plot, phenotypic and genotypic correlation coefficients, and heat map correlation of phenological traits. 

Concerning molecular markers, the most frequent vectors were SCoT 2 and SCoT 5, which had a positive direction of discriminating traits that showed their better performance. It also results from the graph that ISSR-835, ISSR-857 and ISSR-826 vectors were strongly correlated variables. Thus, PCA analysis enabled the decline of 25 key traits to a few variables, while retaining a significant portion of primary data variance. In a similar manner, Beheshtizadeh et al. [85] reported that PCA revealed that 4 components accounted for approximately 76% of the total variation of wheat genotypes traits. Furthermore, Adilova et al. [86] discovered that the PCA revealed four principal components (PC) with eigenvalues greater than one, indicating approximately 90.8% of the total variability. 

### 3.4. DNA Barcoding: Plastid rbcL and matK loci Sequencing

The interpretation of plastid DNA sequences in many plants revealed that the plastid genome changed only possibly a bit during evolutionary events [87,88]. In many studies, the *rbcL* gene has been successfully utilized in phylogenetic studies at above-species taxonomic levels [89,90]. Most studies have emphasized the importance of using *rbcL* in conjunction with other plant DNA barcodes [91]. Furthermore, the *matK* gene is a useful region because it is the fastest evolving plastid gene, providing enough information to identify phylogenies [92]. The Consortium for the Barcode of Life (CBOL) has established the *rbcL* and *matK* as standard plant barcodes, with high species identification and ideal sequential efficiency for plants [87,93]. 

In the present study, the recorded amplicons were shorter (approximately 600 and 900 bp for *rbcL* and *matK*, respectively), allowing for effective sequencing even though many authors deny the region as a barcode if it is longer than 1000 bp due to difficulties in bi-directional sequencing [87]. Our findings agreed with those of Mohamed et al. [94], who concluded that the *matK* gene is more flexible than the *rbcL* gene among Egyptian barley *(Hordeum vulgare* L.) cultivars, and the documented size of the *matK* region’s PCR product was 900 bp, whereas the *rbcL* region was 600 bp.

**Figure 6:**
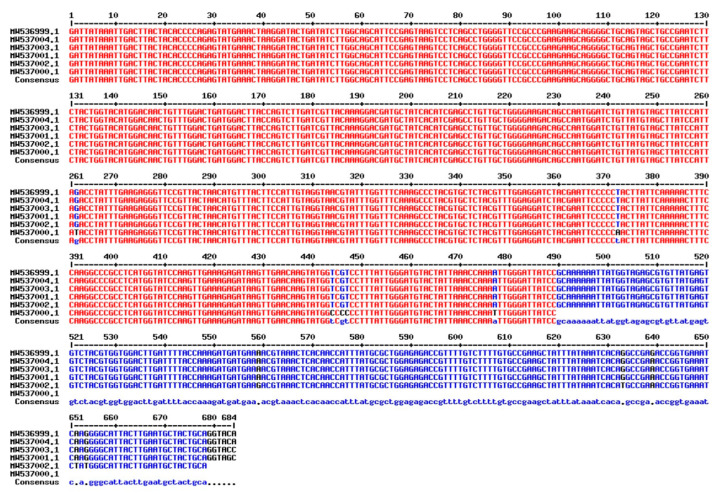
Sequence alignment of *rbcL* gene of the selected six *T. aestivum* genotypes using Multalin ver. 5.4.1 (http://multalin.toulouse.inra.fr/multalin/, accessed on 15 August 2022).

**Figure 7:**
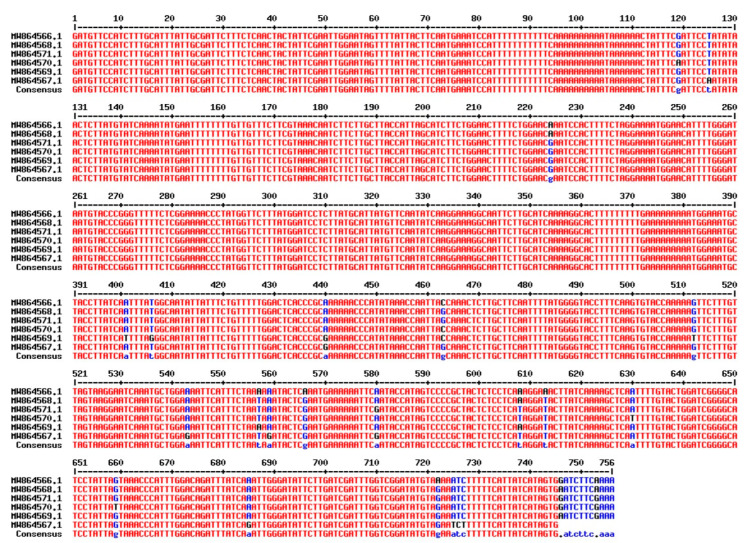
Sequence alignment of *matK* gene of the selected six *T. aestivum* genotypes using Multalin ver.5.4.1 (http://multalin.toulouse.inra.fr/multalin/, accessed on 15 August 2022).

After successfully amplifying a portion of the *rbcL* gene, the amplicons were sequenced and deposited in GenBank under accession numbers in six *T. aestivum* genotypes: W16 (Misr3), W3 (SIDS 14), W7 (GIZA 171), W4 (SHANDWEEL 1), W13 (GEMMIZA 12) and W6 (SAKHA 95) are MW537004, MW536999, MW537003, MW537001, MW537002 and MW537000. In addition, the Genebank accession number for *matK* region in six *T. aestivum* genotypes: W16 (Misr3), W3 (SIDS 14), W7 (GIZA 171), W4 (SHANDWEEL 1), W13 (GEMMIZA 12) and W6 (SAKHA 95) are MW864566, MW864570, MW864567, MW864571, MW864568 and MW864569, respectively. A BLAST function was used to confirm the correct amplification of the *matK* and *rbcL* sequences, which revealed that all of the sequences were strongly coordinated with the *matK* and *rbcL* sequences from *T. aestivum*. The conserved *matK* and *rbcL* gene sequences were used to calculate and evaluate pairwise distances. More information on the *matK* and *rbcL* DNA barcoding regions in six Egyptian *T. aestivum* genotypes is provided in (Figure 6 and Figure 7, respectively).

The genetic variation estimates of DNA barcoding regions of *matK* and *rbcL* loci in 6 Egyptian wheat genotypes were given in Table 8. The length of the amplified *rbcL* gene was approximately 728 bp and 757 bp for *matK* (partial gene) in all studied genotypes. The number of variable sites was 13 and 24 with proportions of 0.018 and 0.032 for *rbcL* and *matK*, respectively. Similarly, the *matK* region was characterized by the greatest number of parsimony informative sites (PICs), 8 with its proportion 0.011 were observed. The average GC content was 0.433 for *rbcL* and 0.316 for *matK* and AT content average was 0.567 and 0.684 for *rbcL* and *matK*, respectively. In the same context, Osman and Ramadan [95] discovered that the length of the amplified *matK* gene in all studied samples was approximately 454 bp (partial gene). The average GC% content in all *Triticum* species was found to be approximately 35.3%.

**Figure 8:**
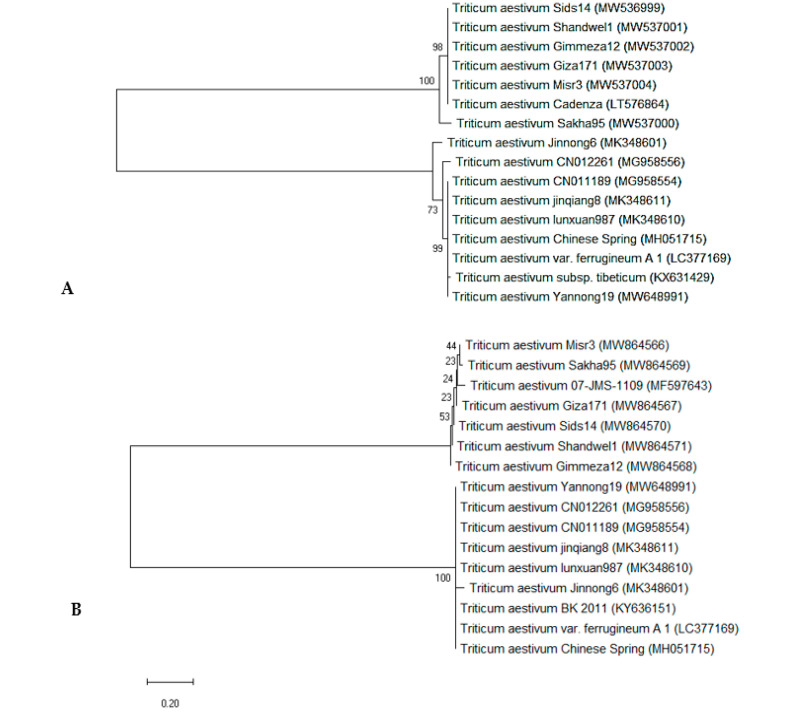
Maximum likelihood tree analysis UPGMA of 6 selected *T. aestivum* genotypes: W16 (MISR 3), W3 (SIDS 14), W7 (GIZA 171), W4 (SHANDWEEL1), W13 (GEMMIZA 12) and W6 (SAKHA 95) and 10 NCBI outgroups, using MEGAX software based on (**A**) *rbcL* sequence data of and (**B**) *matK* sequence data. Numbers indicated on the similarity percentage and the organism accession numbers represented in parentheses.

The phylogenetic tree was built using the neighbor-joining method, and evolutionary distances were calculated using the maximum composite likelihood method. In the *rbcL* gene region (Figure 8A), the six genotypes and 10 *rbcL* sequences, obtained from NCBI and used as outgroups, were distributed into two clusters: the first cluster included two sub-clusters: sub-cluster I had *T. aestivum* candenza (LT576864) NCBI outgroup, and the five wheat genotypes except W6 (SAKHA 95) genotype, which grouped alone in sub-cluster II. Cluster 2 also had two sub-clusters, one contained *T. aestivum* Jinnong6 (MK348601) and the other sub-cluster had the rest of the eight NCBI outgroups. Figure 8B depicts a phylogenetic tree of *matK* sequence variation using the UPGMA algorithm to distinguish between the 6 genotypes studied and 10 *matK* outgroups. The tree has two major clusters: the first included two sub clusters containing the six genotypes and one of the NCBI outgroups with the accession number *T. aestivum* 07-JMS-1109 (MF597643), which is close to both W6 (SAKHA 95) and W7 (GIZA 171) genotypes. Cluster II also had two sub-clusters, one had only one of the NCBI outgroups *T. aestivum* Jinnong6 (MK348601). The second sub-cluster comprises all the rest of the eight NCBI outgroups. In the same trend, Burgess et al. [96] found that the *rbcL*+ *matK* barcode system could identify 93.1% of taxonomic groups sampled from native plants. On the other hand, Awad et al. [97] demonstrated that the core-barcode genes, 6 *matK* and 15 *rbcL*, have limited discrimination capacity of the 18 different Triticum plants in Egypt. Taken together, the use of the two barcodes was ideal for differentiating between different Triticum species in terms of overall barcode system performance and all examined parameters. The best single candidate barcode was *matK*. Because hybridization and mutation are unavoidable, the discovery of efficient barcodes necessitates well-coordinated efforts. This is consistent with the findings of Osman and Ramadan [95], who discovered that the *matK* gene sequence plays an important role in distinguishing between closely related *Triticum* species. As a result, these sequences could be used as a DNA barcode to track the evolution of *Triticum* species. 

## 4. Conclusions

Significant differences in morphological and phenological traits indicated a high level of genetic variability in wheat genotypes, which could be used in future breeding programs for wheat genotype improvement and development. Furthermore, the high polymorphism percentage obtained by ISSR and SCoT demonstrates the power of SCoT as the best in fingerprinting and diversity analyses. The *matK* gene barcode plays an important role in distinguishing among closely related Egyptian *T. aestivum* genotypes. Overall, the combination of desirable phenological parameters with molecular markers and barcodes would be convenient for monitoring Egyptian *T. aestivum* genotypes. As a result, these genotypes and traits are deserving of more attention in future breeding programs aimed at improving wheat.

## Figures and Tables

**Table 1:** Name, pedigree, selection history and year of release of the wheat genotypes and lines analyzed in the present study.

Code	Name	Pedigree	Selection History	Year of Release
W1	SIDS 1	HD 2172/Pavon “S”//1158.57/Maya 74 “S”	S 46-4SD-2SD-1SD-0SD-0EGY	1994
W2	SIDS 12	BUC//7C/ALD/5/MAYA74/ON//1160.147/3/BB/GLL/4/CHAT”S”/6/MAYA/VUL//CMH74A.630/4*SX	SD7096-4SD-1SD-1SD-0SD-0EGY	2009
W3	SIDS 14	BOW “S”/VEE”S”//BOW”S”/TSI/3/BANI SEWEF 1	SD293-1SD-2SD-4SD-0SD-0EGY	-
W4	SHANDWEEL 1	SITE/MO/4/NAC/TH.AC//3*PVN/3/MIRLO/BUC	CMSS93B00567S-72Y-010M-010Y-010M-3Y-0M-0HTY-0SH-0EGY	2011
W5	SAKHA 94	OPATA/RAYON//KAUZ	CMBW90Y3180-0TOPM-3Y-010M-010M-010Y-10M-015Y-0Y-0AP-0S-0EGY	2004
W6	SAKHA 95	PASTOR//SITE/MO/3/CHEN/AEGILOPS SQUARROSA (TAUS)//BCN/4/WBLL1.	CMA01Y00158S-040POY-040M-030ZTM-040SY-26M-0Y-0SY-0S-0EGY	-
W7	GIZA 171	SAKHA 93/GEMMIZA 9	S.6-1GZ-4GZ-1GZ-2GZ-0EGY	2013
W8	GIZA 168	MRL/BUC//SERI	CM93046-8M-0Y-0M-2Y-0B-0SH-0EGY	1999
W9	GEMMIZA 7	CMH 74A.630/5X//SERI 82/3/AGENT	GM 4611-2GM-3GM-1GM-0GM-0EGY	2000
W10	GEMMIZA 9	ALD “S”/HUAC//CMH 74A. 630/5X	GM 4583-5GM-1GM-0GM-0EGY	2000
W11	GEMMIZA 10	MAYA 74 “S”/ON//1160-147/3/BB/GLL/4/CHAT”S”/5/CROW “S”	CGM5820-3GM-1GM-2GM-0GM-0EGY	2004
W12	GEMMIZA 11	BOW”S”/KVZ”S”//7C/SER182/3/GIZA168/SAKHA61	GM7892-2GM-1GM-2GM-1GM-0GM-0EGY	2011
W13	GEMMIZA 12	OTUS/3/SARA/THB//VEE	CMSS97Y00227S-5Y-010M-010Y-010M-2Y-1M-0Y-0GM-0EGY	2013
W14	MISR 1	OASIS/SKAUZ//4*BCN/3/2*PASTOR	CMSS00Y01881T-050M-030Y-030M-030WGY-33M-0Y-0EGY	2014
W15	MISR 2	SKAUZ/BAV92	CMSS96M03611S-1M-010SY-010M-010SY-8M-0Y-0EGY	2014
W16	MISR 3	ATTILA*2/PBW65*2/KACHU	CMSS06Y00582T-099TOPM-099Y-099ZTM-099Y-099M-10WGY-0B-0EGY	2019

**Table 2:** List of primers and their sequences for molecular markers (ISSR and SCoT) and plastid *rbcL* and *matK* genes.

Primer Code	Sequence (5′-3′)	Size (bp)
ISSR marker		
ISSR-807	(AG)8 T	172–1156
ISSR-810	(GA)8 T	159–2949
ISSR-835	(Ag)8 YC	96–4039
ISSR-841	(GA)8 YC	102–420
ISSR-857	(AC)8 YG	84–244
ISSR-825	(AC)7 T	327–2562
ISSR-814	(CT)7 CAT	215–496
ISSR-826	(AC)8 C	223–1808
ISSR-827	(AC)8 G	255–1408
ISSR-840	(gA)8 TT	123–1164
SCoT marker		
SCoT 1	CAACAATGGCTACCACCC	513–3099
SCoT 2	ACCATGGCTACCACCGGC	296–3658
SCoT 3	CAACAATGGCTACCACGC	183–2079
SCoT 4	CAACAATGGCTACCACCG	177–3009
SCoT 5	ACGACATGGCGACCACGC	142–669
SCoT 6	CCATGGCTACCACCGCAG	331–2274
Plastid *rbcL* and *matK* genes		
*rbcL*	F: 5′-ATGTCACCACAAACAGAGACTAAAGC-3′	600
	R: 5′-TCGCATGTACCTGCAGTAGC-3′	
*matK*	F: 5′-CGATCTATTCATTCAATATTTC-3′	900
	R: 5′-TCTAGCACACGAAAGTCGAAGT-3′	

F: Forward primer and R: Reverse primer.

**Table 3:** Descriptive statistics of 16 days seedling, shoot leaf and root morphometric traits and seed geometric traits of the 16 *T. aestivum* genotypes studied.

Morphometric and Geometric Traits	Average	Max.	Min.
Seedling parameters			
PB	0.12	0.20	0.07
Shoot parameters			
SL	14.73	18.26	7.90
LFI	3.15	5.16	1.12
DFI	0.18	0.23	0.15
Leaf Parameters			
LA	2.92	4.48	1.13
LW	0.32	0.42	0.24
Root parameters			
RN	4.19	6.00	3.00
RL	8.60	11.09	6.83
RW	7.03	10.23	5.07
TA	35.89	48.63	27.34
Seed parameters			
SW	0.05	0.06	0.03
SA	0.34	0.45	0.29
SP	2.45	2.81	2.27
L	0.89	1.04	0.82
W	0.49	0.61	0.43
AR	1.84	2.03	1.55
Circ.	0.72	0.75	0.69
Round	0.55	0.66	0.49
Feret	0.90	1.04	0.84

PB = plant biomass (g); SL = shoot length (cm); LFI = length of first internodes (cm); DFI = diameter of first internode (cm); LA = leaf area (cm^2^); LW = leaf width (cm); RN = root number; RL = root maximum length (cm); RW = root width (cm); TA = tip angle of root (°); SW = single seed weight (g); SA = seed area (cm^2^); SP = seed perimeter; L = length of seed major axis (cm); W = length of seed minor axis (cm); AR = aspect ratio; Circ. = circulatory; Round = roundness; Feret = Feret diameter (cm); Average = Mean; Min. = Minimum; Max. = Maximum.

**Table 4:** Estimation of genotypic coefficient variance, heritability analysis of morphometric traits of the 16 *T. aestivum* genotypes studied.

TRAITS	R MS (2)	G MS (15)	RXG MS (30)	RCV%	GCV%	RXG CV (%)	H2%	LSD (5%)	F VALUE OF G
**PB**	0.0015	0.0032	0.0011	0.0001	0.007	0.0003	65.33	0.06	2.88 **
**SL**	3.1912	24.0514	2.7140	0.0298	7.1125	0.6785	88.72	2.75	8.86 **
**LFI**	0.0889	2.2307	0.2609	0.0057	0.6566	0.0652	88.30	0.85	8.55 **
**DFI**	0.002	0.0014	0.0014	0.0000	0.0000	0.0003	4.93	0.06	1.05
**LA**	0.0777	2.7584	0.3378	0.0063	0.8069	0.0844	87.76	0.97	8.17 **
**LW**	0.0125	0.0079	0.0058	0.0004	0.007	0.0014	26.91	0.13	1.37
**RN**	0.000	2.4875	0.000	0.0000	0.8292	0.0000	100.00	0.00	0.00
**RL**	2.8541	4.8841	1.6033	0.0782	1.0936	0.4008	67.17	2.11	3.05 **
**RW**	1.2819	6.6247	4.2020	0.0867	0.8076	1.0505	36.57	3.42	1.58
**TA**	164.8038	93.6098	33.5567	8.2029	20.0177	8.3892	64.15	9.66	2.79 **
**SW**	0.000	0.0002	0.0001	0.0000	0.0001	0.000	74.02	0.00	0.00
**SA**	0.0006	0.0060	0.0010	0.0000	0.0017	0.0002	83.77	0.05	6.16 **
**SP**	0.0024	0.0638	0.0089	0.0002	0.0183	0.0022	86.07	0.16	7.18 **
**L**	0.0000	0.0082	0.0013	0.0000	0.0023	0.0003	84.78	0.06	6.57 **
**W**	0.0010	0.0052	0.0013	0.0000	0.0013	0.0003	74.08	0.06	3.86 **
**AR**	0.0099	0.0434	0.0178	0.0005	0.0086	0.0044	59.11	0.22	2.45 *
**CIRC.**	0.0001	0.0005	0.0002	0.0000	0.0001	0.0001	58.82	0.02	2.43 *
**ROUND**	0.0010	0.0047	0.0021	0.0001	0.0009	0.0005	56.11	0.08	2.28 *
**FERET**	0.0001	0.0083	0.0011	0.0000	0.0024	0.0003	86.68	0.06	7.51 **

R = Replications; G = Genotypes; RXG = Replication with genotypes; Parenthesis indicate degree of freedom; MS = Mean square; RCV% = Replication Coefficient of Variance; GCV% = Genotypic Coefficient of Variance; H^2^ (%) = heritability, LSD (5%) = Least significant differences, F value of = F value of genotypes, *, ** significant at the 0.05 and 0.01 level of the probability, respectively

**Table 5:** Genotypic (r_g_) (lower diagonal) and phenotypic (r_p_) (above diagonal) correlation matrix among all morphological traits of the 16 *T. aestivum* genotypes studied.

Parameters	PB	SL	LFI	DFI	LA	LW	RN	RL	RW	TA	SW	SA	SP	L	W	AR	Circ.	Round	Feret
PB	0	0.21	0.41	0.05	0.47	0.39	0.14	0.11	0.4	0.26	0.57 *	0.72 **	0.75 **	0.74 **	0.55 *	0.04	−0.07	0.03	0.76 **
SL	0.23	0	0.73 **	0.35	0.80 **	0.4	−0.67 **	0.25	0.02	0.02	−0.01	0.17	0.19	0.19	0.14	0.01	−0.02	0.03	0.19
LFI	0.50 +	0.78 +	0	0.07	0.66 **	0.39	−0.42	0.11	−0.21	−0.02	0.21	0.53 *	0.48	0.3	0.59 *	−0.37	0.35	0.44	0.44
DFI	−0.22	1.36	−0.49	0	0.36	0.26	−0.02	−0.16	0.49	0.01	−0.19	−0.43	−0.4	−0.28	−0.44	0.28	−0.29	−0.26	−0.37
LA	0.56 ++	0.84 ++	0.75 ++	1.74	0	0.75 **	−0.45	0.17	0.26	−0.1	−0.06	0.45	0.46	0.44	0.37	−0.05	0.04	0.1	0.47
LW	0.64 +	0.83 +	0.85 +	1.24	1.51 +	0	−0.12	−0.02	0.35	−0.37	−0.06	0.45	0.41	0.29	0.45	−0.29	0.28	0.31	0.38
RN	0.17	−0.72 ++	−0.44 ++	−0.1	−0.48 ++	−0.24	0	−0.61 *	0.22	−0.09	0.18	0.04	0.02	−0.03	0.08	−0.08	0.07	0.11	0.01
RL	0.3	0.30 +	0.18	−0.02	0.18	0.31	−0.74 +	0	−0.03	0.41	0.13	0.14	0.18	0.26	0.02	0.21	−0.21	−0.19	0.21
RW	0.75 +	−0.09	−0.48 +	3.02	0.43 +	0.45	0.37	0.05	0	−0.14	−0.04	0.03	0.1	−0.27	0.15	0.39	−0.4	−0.37	0.16
TA	0.48 +	0.05	0.06	0.31	−0.16	−0.82 +	−0.11	0.55 +	0	0	0.17	−0.07	−0.03	−0.09	0.19	0.36	−0.37	−0.31	0.01
SW	0.88 ++	−0.04	0.26	−0.9	−0.13	−0.25	0.21	0.19	−0.1	0.18	0	0.45	0.45	0.38	0.41	−0.11	0.11	0.13	0.44
SA	1.07 ++	0.24	0.62 ++	−2.09	0.55 ++	0.76 +	0.05	0.17	0.03	−0.1	0.51 +	0	0.99 **	0.82 **	0.92 **	−0.35	0.33	0.4	0.95 **
SP	1.07 ++	0.25	0.57 ++	−1.92	0.54 ++	0.69 +	0.02	0.21	0.14	−0.05	0.50 +	0.99 ++	0	0.90 **	0.84 **	−0.19	0.17	0.24	0.99 **
L	1.01 ++	0.23	0.37 +	−1.3	0.48 ++	0.51 +	−0.04	0.29	0.44 +	0.11	0.40 +	0.88 ++	0.93 ++	0	0.53*	0.24	−0.26	−0.2	0.95 **
W	0.92 ++	0.23	0.72 ++	−2.31	0.52 ++	0.79 +	0.09	0.06	−0.29	−0.26	0.52 +	0.93 ++	0.87 ++	0.64 ++	0	−0.69 **	0.67 **	0.73 **	0.76 **
AR	−0.05	−0.03	−0.47	1.65 +	−0.19	−0.59	−0.11	0.22	0.76 +	0.53 +	−0.22	0.31 +	−0.18	−0.18	0.64 ++	0	−1.00 **	−0.99 **	−0.05
Circ.	0	0.02	0.44 +	−1.66	0.17	0.56	0.09	−0.22	−0.76 +	−0.55 +	0.21	0.29	0.16	−0.21	0.62 ++	−1.00 ++	0	0.98 **	0.03
Round	0.21	0.08	0.57 ++	−1.66	0.25	0.62	0.15	−0.23	−0.76 +	−0.48 +	0.24	0.38 +	0.25	−0.12	0.69 ++	−1.00 ++	1.00 ++	0	0.11
Feret	1.07 ++	0.25	0.52 ++	−1.74	0.53 ++	0.64 +	0.01	0.24	0.24	0.01	0.47 +	0.97 ++	0.99 ++	0.97 ++	0.81 ++	−0.06	0.04	0.13	0

Green box: *, ** significant at the 0.05 and 0.01 level of the probability, respectively. Blue box: +, ++ coefficient of correlation is larger than one and two times the standard error, respectively.

**Table 6:** Number of amplified bands, number of polymorphic bands, polymorphism percentage, mean of band frequency and marker efficiency calculations of the 16 ISSR and SCoT markers assayed for the 16 *T. aestivum* genotypes studied.

Molecular Marker	MB	Polymorphic Bands			TAB	PPB	MBF	H	PIC	E	MI	H.av	D	Rp
		UB	NUB	NPB										
ISSR-807	1	7	0	7	8	88	0.5	0.50	0.37	3.8	0.02	0.00	0.78	2.9
ISSR-810	2	6	0	6	8	75	0.6	0.49	0.37	4.5	0.02	0.00	0.69	3.3
ISSR-835	4	5	2	7	11	64	0.7	0.44	0.34	7.4	0.02	0.00	0.55	3.6
ISSR-841	3	3	1	4	7	57	0.5	0.49	0.37	3.9	0.02	0.00	0.70	1.7
ISSR-857	0	9	1	10	10	100	0.3	0.44	0.35	3.3	0.01	0.00	0.89	4.9
ISSR-825	1	5	0	5	6	83	0.5	0.50	0.37	3.1	0.02	0.01	0.73	2.7
ISSR-814	0	2	1	3	3	100	0.3	0.39	0.31	0.8	0.01	0.01	0.93	1.2
ISSR-826	3	7	0	7	10	70	0.8	0.35	0.29	7.7	0.02	0.00	0.40	2.4
ISSR-827	1	5	2	7	8	88	0.5	0.50	0.37	3.7	0.02	0.00	0.78	2.3
ISSR-840	0	7	1	8	8	100	0.3	0.39	0.31	2.1	0.01	0.00	0.93	4.1
Total	15	56	8	64	79	-	-	4.50	3.47	40.4	0.15	0.04	7.38	29.2
Mean	1.5	5.6	0.8	6.4	7.9	82.41	0.49	0.45	0.35	4.04	0.01	0.00	0.74	2.92
SCoT 1	0	8	0	8	8	100	0.3	0.40	0.30	2.6	0.01	0.00	0.9	3.3
SCoT 2	2	9	0	9	11	82	0.6	0.50	0.40	6.3	0.02	0.00	0.7	5.1
SCoT 3	1	9	1	10	11	91	0.3	0.50	0.40	3.9	0.01	0.00	0.9	5.7
SCoT 4	0	11	2	13	13	100	0.3	0.40	0.30	3.5	0.01	0.00	0.9	6.5
SCoT 5	0	10	0	10	10	100	0.4	0.50	0.40	4.5	0.01	0.00	0.8	7.1
SCoT 6	0	7	4	11	11	100	0.2	0.40	0.30	2.8	0.01	0.00	0.9	4.9
Total	3	54	7	61	64	-	-	2.70	2.10	23.6	0.07	0.02	5.1	32.7
Mean	0.5	9	1.17	10.17	10.70	95.46	0.36	0.40	0.30	3.9	0.01	0.00	0.9	5.4
Total of All	18	110	15	125	143	-	-	7.2	5.5	64.0	0.21	0.06	12.5	61.9
Mean of All	1.2	7.33	1	8.33	9.53	88.94	0.43	0.4	0.3	4.0	0.01	0.00	0.8	3.9

MB, Monomorphic bands; UB, unique bands; NUB, non-unique bands; NPB, number of polymorphic bands; TAB, total amplified bands; PPB, percentage of polymorphic bands; MBF, mean of band frequency; H, heterozygosity index; E, effective multiplex ratio; PIC, polymorphism information content; H.av, average expected heterozygosity; D, discriminating power; MI, marker index; Rp, resolving power.

**Table 7:** Genetic similarity of 16 *T. aestivum* genotypes by Jaccard’s coefficient using UPGMA algorithm based on ISSR and SCoT banding patterns.

Genotypes	W1	W2	W3	W4	W5	W6	W7	W8	W9	W10	W11	W12	W13	W14	W15	W16
W1	1.00															
W2	0.79	1.00														
W3	0.87	0.75	1.00													
W4	0.73	0.69	0.74	1.00												
W5	0.81	0.79	0.82	0.68	1.00											
W6	0.60	0.63	0.61	0.51	0.71	1.00										
W7	0.71	0.67	0.71	0.67	0.69	0.71	1.00									
W8	0.88	0.85	0.86	0.78	0.88	0.63	0.73	1.00								
W9	0.86	0.77	0.79	0.69	0.83	0.64	0.72	0.83	1.00							
W10	0.83	0.79	0.84	0.66	0.76	0.64	0.73	0.83	0.79	1.00						
W11	0.79	0.78	0.80	0.74	0.82	0.68	0.79	0.81	0.77	0.76	1.00					
W12	0.80	0.73	0.80	0.63	0.77	0.73	0.79	0.81	0.73	0.89	0.78	1.00				
W13	0.71	0.72	0.67	0.56	0.72	0.67	0.65	0.73	0.72	0.72	0.72	0.78	1.00			
W14	0.76	0.66	0.77	0.71	0.68	0.56	0.62	0.78	0.72	0.66	0.68	0.71	0.64	1.00		
W15	0.73	0.69	0.72	0.64	0.64	0.69	0.78	0.71	0.67	0.74	0.72	0.76	0.65	0.67	1.00	
W16	0.73	0.74	0.76	0.63	0.80	0.77	0.75	0.77	0.74	0.75	0.78	0.79	0.79	0.68	0.69	1.00

**Table 8:** Genetic variation estimates of DNA barcoding of 2 chloroplast (*matK* and *rbcL)* genes of 16 *T. aestivum* genotypes.

Barcode/Genetic Variability of each Marker	*rbcL*	*matK*
Total alignment length (bp)	728	757
Total matrix cells	4368	4542
Missing percent	9.501	0.132
Number of variable sites	13	24
Proportion of variable sites	0.018	0.032
Number of parsimony informative sites (PIC)	0.00	8
Proportion of Parsimony informative sites	0.000	0.011
AT content	0.567	0.684
GC content	0.433	0.316
A	1112	1429
C	821	795
G	891	641
T	1129	1671

A, Adenine; C, Cytosine; G, Guanine and T, Thymine.

## Data Availability

All the data supporting this study are included in this manuscript.

## Abbreviations

PB: plant biomass; SL, shoot length; LFI, length of first internodes; DFI, diameter of first internode; LA, leaf area; LW, leaf width; RN, root number; RL, root maximum length; RW, root width; TA, tip angle of root (°); SW, single seed weight; SA, seed area; SP, seed perimeter; L, length of seed major axis; W, length of seed minor axis; AR, aspect ratio; Circ., circulatory; Round, roundness; Feret, Feret diameter; R, Replications; G, Genotypes; RXG, Replication with genotypes; Genotypic Coefficient of Variance; RCV% = Replication Coefficient of Variance, GCV%; H^2^ (%), heritability; LSD (5%), Least significant differences; rg, Genotypic correlation coefficients; rp, Phenotypic correlation coefficients; NPB, Number of polymorphic bands; MB, Monomorphic bands; NUB, Non-unique bands; UB, Unique bands; TAB, Total amplified bands; MBF, Mean of band frequency; PIC, Polymorphism information content; PPB, Percentage of polymorphic bands; H, Heterozygosity index; E, Effective multiplex ratio; MI, Marker index; H.av, Average expected heterozygosity; Rp, Resolving power; D, Discriminating power; *matK*, maturase K; *rbcL*, ribulose-1, 5-bisphosphate carboxylase oxygenase; ML, Maximum likelihood and NJ, Neighbor joining.
